# Identifying Genetic Factors Contributing to Non-Syndromic Early-Onset Childhood Obesity Utilizing Whole-Exome Sequencing in Consanguineous Families

**DOI:** 10.3390/genes17050530

**Published:** 2026-04-29

**Authors:** Hazal Banu Olgun Celebioglu, Ayse Pinar Ozturk, Sukran Poyrazoglu, Feyza Nur Tuncer

**Affiliations:** 1 Department of Genetics, Aziz Sancar Institute of Experimental Medicine, Istanbul University, 34093 Istanbul, Türkiye; hbcelebioglu@gmail.com; 2 Graduate School of Health Sciences, Istanbul University, 34126 Istanbul, Türkiye; 3 Pediatrics Endocrinology Unit, Department of Pediatrics, Istanbul Faculty of Medicine, Istanbul University, 34093 Istanbul, Türkiye; ozturk.aysep@gmail.com (A.P.O.); sukran.poyrazoglu@istanbul.edu.tr (S.P.)

**Keywords:** early-onset obesity, whole-exome sequencing (WES), obesity genetics, novel variants, body mass index (BMI)

## Abstract

Purpose: Obesity, characterized by abnormal fat accumulation with comorbidities, continues to increase dramatically, particularly in the pediatric population. Identifying the environmental and genetic causes underlying the development of obesity during early childhood is crucial for establishing preventive and protective treatments for this complex disease. We aimed to investigate genetic variants related to non-syndromic early-onset childhood obesity. Methods: Whole-exome sequencing was performed in three independent consanguineous families with obesity, including three index cases and two additional affected siblings. Non-synonymous variants with minor allele frequency < 0.01 in all normal populations were filtered using the Genomize-SEQ Platform. Variant confirmations and familial segregations were analyzed by Sanger sequencing. Results: WES revealed a shared *ATXN3* gene variant and two known variants of the *SH2B1* and *ADIPOQ* genes, which were reported to be associated with obesity. Additionally, five heterozygous novel gene variants of the *ANKK1*, *NEGR1*, *OGDH*, *ABCB1*, and *GSK3B* genes were identified, which are predicted to cause excessive fat accumulation and disruption of energy balance in individuals. Conclusions: We suggest that the cumulative effects of all obesity-associated detected variants lead to the early-onset obesity phenotype observed in individuals. Hence, periodic follow-up and treatment opportunities are recommended for index cases, alongside the adoption of a more active lifestyle and healthy nutrition practices.

## 1. Introduction

Obesity is defined by the World Health Organization (WHO) as excessive body fat accumulation that poses significant, life-threatening health risks through the development of multiple comorbidities. This heterogeneous disorder is driven by both environmental and genetic factors, occurs more frequently in preschool children and adolescents than in adults, tends to persist throughout life once established, and is increasingly recognized as a state of chronic low-grade systemic inflammation contributing to its long-term complications [1,2,3]. Obesity arises from an imbalance between energy intake and expenditure, with individuals exhibiting high body mass index (BMI) values facing an elevated risk of mortality due to complications caused by type 2 diabetes mellitus (T2DM), cardiovascular disease, and various types of cancer [4,5]. Obesity may originate as early as the prenatal and infancy periods, influenced by factors such as birth weight, gestational age, early feeding patterns, malnutrition, rapid postnatal growth, and physical inactivity. Excessive weight gain beginning early in life is strongly associated with childhood obesity, while accelerated growth during infancy contributes to the development of obesity-related comorbidities later in adulthood [6,7,8]. In this context, early-onset obesity refers to obesity developing in early childhood, typically before the age of five, and may arise from disruptions in genes regulating energy balance, appetite, and adipose tissue distribution [9].

This complex disorder may result from genetic variants that predispose individuals to elevated BMI. Genetic delineation of obesity, particularly in early-onset and severe cases, together with targeted genetic testing, is therefore essential for confirming the diagnosis of obesity, defining obesity subtypes, assessing recurrence risk, and providing appropriate genetic counseling for affected families [9,10]. In this regard, whole-exome sequencing (WES) has extensively been utilized in detecting rare variants involved in disease onset [11]. A comprehensive understanding of the genetic and early-life risk factors contributing to the increasing prevalence of early-onset childhood obesity is essential for the development of effective therapeutic and preventive strategies [12,13].

Accordingly, consanguineous families provide a valuable framework for investigating the genetic basis of early-onset obesity. In populations with high rates of consanguinity, large genomic regions are inherited from common ancestors, leading to increased homozygosity and a higher prevalence of rare recessive monogenic disorders as well as complex traits such as obesity [14]. The reduced genetic heterogeneity associated with endogamy further enhances the likelihood of identifying candidate genes. In this regard, the consanguineous marriage rate in Türkiye remains as high as approximately 24%, supporting the inclusion of consanguineous families in the present study [15]. Thereby, this study aimed to identify novel rare coding gene variants associated with early-onset childhood obesity in three distinct consanguineous families by utilizing WES analysis.

## 2. Materials and Methods

### 2.1. Patients and Clinical Evaluations

**Figure 1:**
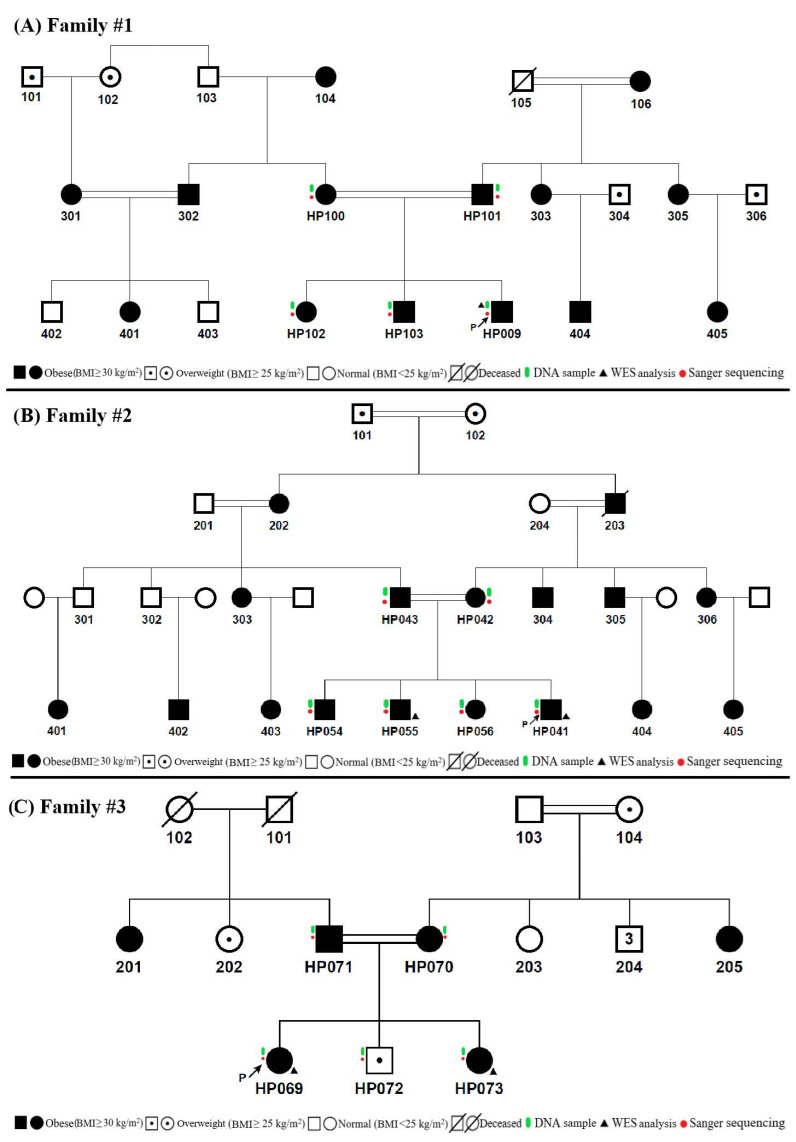
(**A**) Pedigree of family #1. (**B**) Pedigree of family #2. (**C**) Pedigree of family #3. The index cases of each family are shown with “P” on the pedigrees. All the experimental analyses are shown with colored figures beside the subjected family members. Accordingly, genomic DNA samples were taken from individuals marked in green, WES analyses were performed for individuals indicated with black triangles, and individuals marked in red were subjected to familial segregation using the Sanger sequencing method.

Three independent consanguineous families involving individuals affected by obesity were recruited between October 2020 and October 2021 from a single childhood endocrinology clinic in Türkiye for this study. Detailed family history interviews were conducted with the parents during recruitment to assess consanguinity. Based on parental reports, Families 1 and 3 were identified as having shared great-grandparents, whereas Family 2 was reported to have shared grandparents (Figure 1). The inclusion criteria required families to have at least two children with early-onset excessive weight gain beginning before the age of five and in the absence of any syndromic obesity features like intellectual disability, dysmorphic characteristics, or organ-specific developmental anomalies. All index cases were subjected to detailed physical and clinical examinations by physicians, and detailed information on family history was collected from adult recruits. Weight and height measurements were taken without clothing or shoes from the index cases and their siblings. The growth chart prepared with national standards were followed to calculate BMI and standard deviation scores (SDSs) for children (age < 18) and weight for height SDSs according to WHO growth standards were used for children younger than two years of age [16,17]. Standard BMI calculations were completed using the weight and height features of adult family members based on their oral declarations. As instructed by WHO growth standards, BMI values ≥ 30 kg/m^2^ for adults and ≥2 SDS for children were accepted as obese [18]. Clinical evaluations of the recruited relatives of the index cases were based on medical records obtained from routine clinical and laboratory assessments. Written informed consents were obtained from all involved family members or their legal representatives, and all analyses were implemented after the approval in compliance with the Istanbul Medical Faculty Clinical Research Ethics requirements, Istanbul University (protocol no: 2020/1054).

### 2.2. Genetic and Bioinformatic Analyses

Peripheral blood samples were collected from all index cases and available family members to obtain genomic DNA, and WES was performed in three independent consanguineous families with obesity, including three index cases (HP009, HP041 and HP069), along with two additional affected siblings (HP055 and HP073). DNA was extracted from peripheral blood using the Purelink Genomic DNA Mini Kit (Invitrogen, Thermo Fisher Scientific, Inc., Waltham, MA, USA), following the manufacturer’s instructions. WES was performed for the selected family members. Protocols for WES analysis, library preparations, and variant calling were adopted from the recently published study by Olgun et al. [9], while the Illumina NextSeq 550 platform was utilized to ensure that the targeted bases had a minimum reading depth of 40×. The SEQ platform version 16.7 (https://seq.genomize.com; Genomize Inc., Istanbul, Türkiye) was utilized for sequence annotations and variant filtering, which operated FASTQ files by aligning to the GRCh37/hg19 reference genome with the Burrows–Wheeler Alignment (BWA) tool (version 0.7.18) to generate the final BAM file and variant list, as Olgun et al. mentioned [9].

Variant prioritization was performed for MAF < 1% in all normal populations to detect rare variants, which were obtained from GnomAD (https://gnomad.broadinstitute.org/, accessed on 5 February 2025), the dbSNP database (https://www.ncbi.nlm.nih.gov/SNP/, accessed on 10 February 2025), the 1000 Genomes Project (http://www.1000genomes.org/, accessed on 12 February 2025), the Exome Sequencing Project (ESP6500; provided by the SEQ platform accessed on 20 March 2025), TopMED (https://topmed.nhlbi.nih.gov/, accessed on 26 February 2025), the Greater Middle East Variome Project (GME; https://illumina.github.io/NirvanaDocumentation/data-sources/gme/, accessed on 20 March 2025) and SEQ-specific cohorts comprising approximately 15,000 exome sequences of individuals from Turkiye with varying disorders (accessed on 20 March 2025). A stepwise filtering strategy was adopted. Initially, in order to identify potential common genetic contributors, rare variants shared among all sequenced individuals from the three families were filtered for. During this exploratory step, only non-coding intronic variants without predicted splice relevance were excluded to reduce candidate burden and prioritize variants with a higher likelihood of functional impact. Subsequently, variants identified in previously reported obesity-related genes based on a literature review (Supplementary Table S1) were evaluated regardless of variant type [9]. Variant prioritization at this stage was based on predicted pathogenicity, potential splice effects, previous disease associations, and biological relevance. In the final stage, each family was analyzed separately utilizing family segregation to identify potentially novel candidate variants. Due to the presence of consanguinity, homozygous variants consistent with an autosomal recessive inheritance model were prioritized initially. When no plausible candidate variant was identified under this model, heterozygous variants segregating with the phenotype were subsequently evaluated. Compound heterozygous variants were also reviewed when applicable. Preliminary candidate genes generated from this step-wise prioritization approach were subjected to evaluation for functional relevance to disease pathogenesis through a literature search. Furthermore, a set of *in silico* prediction tools (PolyPhen2, SIFT, Mutation Taster, 1000 Genomes, DANN, CADD) were used to assess candidate variants in addition to the Genomic Evolutionary Rate Profiling (GERP) score, which estimates evolutionary constraints in a specific position. Variant pathogenicity was assessed according to the American College of Medical Genetics (ACMG) guidelines, incorporating SEQ platform-based evaluations. For variant pathogenicity assessment, the SEQ platform integrates multiple parameters, including population frequency databases, in silico prediction tools, literature resources, and internal reference datasets. In addition, variant classifications based on the ACMG guidelines were obtained through the platform output, and variants were categorized as pathogenic, likely pathogenic, variants of uncertain significance (VUS), likely benign, or benign. Sanger sequencing was utilized to validate candidate variants and segregation in the families, where sequences were analyzed using CLC Main Workbench. Afterwards, gene–gene and protein–protein interactions and pathway analyses were investigated using the Kyoto Encyclopedia of Genes and Genomes (KEGG), Search Tool for the Retrieval of Interacting Genes (STRING), and Uniprot database. KEGG pathways with a false discovery rate (FDR) of less than 0.05 were deemed significant.

## 3. Results

### 3.1. Clinical Evaluations of Family #1

Hereby, we describe a family with parents and children having early-onset obesity from a consanguinity marriage (Figure 1A). The BMIs of HP100 (mother) and HP101 (father) were 33.3 kg/m^2^ (Obesity Class I) and 35.5 kg/m^2^ (Obesity Class II), respectively, on admission. The mother, HP100, underwent multiple surgical procedures caused by heart disease, and she had comorbidities of hypertension in addition to hyperlipidemia, while her parents suffered from diabetes mellitus (DM), hypertension, and heart disease. Index’s sister, HP102, had a BMI of 32.9 kg/m^2^ (Obesity Class I) and insulin resistance. Index’s brother, HP103, was obese with a BMI of +2.13 SDS on admission.

The index case, HP009, was delivered at the 36th gestational week by Caesarean section at normal weight with no other complications. Upon presentation to our clinic, his weight and height were measured as 81 kg (+3.45 SDS) and 136 cm (−0.74 SDS), respectively, and his BMI SDS was calculated as +3.64, which defined him as having severe obesity for nine years of age. He had normal neuro-motor development and no dysmorphic features, ruling out syndromic obesity. He had insulin resistance diagnosed at the age of eight and Grade III hepatic steatosis on ultrasonography. He started to gain weight after five years of age. Moreover, all children in this family were reported to exhibit similar growth patterns, characterized by rapid weight gain during early childhood, which persisted over time. Both parents experienced a similar situation, describing themselves and their siblings as obese during their childhood.

### 3.2. Clinical Evaluations of Family #2

Family #2 included four children diagnosed with obesity, born to consanguineous parents (Figure 1B). The BMIs of HP042 (mother) and HP043 (father) were 37.8 kg/m^2^ (Obesity Class II) and 36.5 kg/m^2^ (Obesity Class II), respectively. The siblings of HP041 (index case) also had obesity, where HP054’s BMI was 38.1 kg/m^2^ (Obesity Class II), HP055’s BMI was 42.2 kg/m^2^ (Obesity Class III), and HP056’s BMI was 35.4 kg/m^2^ (Obesity Class I). Several metabolic disorders considered to be consequences of obesity were identified in the family, as follows: DM in the father HP043, hypertension in the oldest brother HP054, and nephropathy in the sister HP056.

The index case, HP041, was referred to endocrine clinics at the age of 11 years with a weight of 76.4 kg (+2.81 SDS), a height of 152 cm (+0.96 SDS), and +2.8 BMI SDS. He was born at term by Caesarean section at standard weight with no other dysmorphic features. He was breastfed for two years and started gaining weight around the age of five years, just like his siblings, as stated by his parents. His body fat composition was enhanced by the beginning of primary school and exhibited an accelerated pattern each year. He had insulin resistance and Grade I hepatic steatosis on ultrasonography.

### 3.3. Clinical Evaluations of Family #3

This family was composed of three children with obesity (Figure 1C). The BMIs of the parents were calculated as 31.6 kg/m^2^ (Obesity Class I) for the mother (HP070) and 33.4 kg/m^2^ (Obesity Class I) for the father (HP071). The parents did not declare any other comorbidities. The 17-year-old index case, HP069, was reportedly born at term with a normal birth weight and was breastfed for two years. She was reported to have been overweight during childhood, with rapid weight gain beginning after puberty. Her body measures were 98 kg (+4.34 SDS) for weight, 161 cm (−0.32 SDS) for height, and +3.72 BMI SDS on admission. She had no syndromic features. Moreover, the 11-year-old son, HP072, was classified as overweight (BMI SDS +1.8), while the eight-year-old daughter was classified as obese (BMI SDS +2.24).

### 3.4. Whole-Exome Sequencing Results

Table 1 summarizes quality parameters and filtering results for WES regarding each family under study, while Table 2, Table 3 and Table 4 represent the selected candidate variants in accordance with our three-step strategy.

The variant rs193922928 of *ATXN3* was found to be shared by all affected members among the three families (Table 2). Upon conducting further analysis of known variants, two single-nucleotide polymorphisms (SNPs) were identified in the *ADIPOQ* and *SH2B1* genes in families #2 and #3, respectively, both of which have been reported to be associated with obesity-related traits and were classified as likely pathogenic based on ACMG criteria (Table 3). Additionally, four novel missense variants and one novel stop-gained variant were identified for the obesity-related genes, which were predicted to be deleterious by Mutation Taster and were classified as variants of uncertain significance (VUS) according to ACMG criteria (Table 4). All identified variants were carefully assessed as potential candidates warranting further investigation in the context of obesity, except for those that had been previously reported.

## 4. Discussion

Obesity is an escalating health problem and a major risk factor for severe comorbidities. Influenced by genetic and environmental risk factors, its prevalence is rising sharply, especially among pediatric populations, underscoring concerns about long-term impacts, including adult obesity [19]. Hence, regular clinical follow-ups are essential for effective weight management in children and adolescents, particularly those showing early-onset excessive weight gain patterns who would benefit from early interventions [20]. According to recent data, 61% of the Turkish population is categorized as overweight, with approximately 32.1% classified as living with obesity [21]. Therefore, working with three independent consanguineous families having multiple affected members has been an indispensable resource in determining candidate obesity genes in this understudied population.

### 4.1. A Genetic Variant Shared Among the Families

The Ataxin 3 (*ATXN3*) p. (Gln297_Gln305dup) (rs3356231) inframe insertion variant was found in all index cases among the three families. While HP009 (Family #1) is homozygous, the others are heterozygous for this variant. This gene encodes a protein that has (CAG)n repeats in the coding region and is involved in the primary cysteine deubiquitinating enzyme family [22]. CAG repeat length variants in polyglutamine disease-associated genes (PDAGs), including the *ATXN3* gene, cause various neurodegenerative disorders [23]. The expanding of CAG repeats beyond 52 was found to generate extended proteins, leading to misfolding of the protein to form aggregates. This “gain of toxic protein function” causes impairment of the proteasomal protein degradation pathway, leading to Machado–Joseph disease (MJD), described as a progressive neurologic ataxia [24]. The p.(Gln297_Gln305dup) variant has been reported as “benign” and “likely benign” in the ClinVar database regarding MJD, which correlates with our cases who do not manifest MJD. Interestingly, it has been determined that CAG repeat length variants in the non-mutant range can be risk factors for neuropsychiatric conditions, which paved the path for its correlation with BMI [19]. In this respect, currently, BMI is not directly associated with the absolute number of CAG repeats in the *ATXN3* gene, but rather with the difference in CAG repeat length between the two alleles. Because the shorter and longer alleles showed opposite directional effects on BMI, the authors focused on the allelic difference as a key determinant. This difference was found to be non-linearly associated with BMI, following a U-shaped (quadratic) relationship, indicating that neither highly similar nor highly divergent allelic CAG repeat lengths produced the most pronounced effects on BMI [19]. In this context, the *ATXN3* (rs3356231) variant shared across families in our cohort exhibited CAG repeat lengths within the non-pathological range. While these repeat lengths are not considered pathogenic, specific allelic combinations, rather than absolute repeat size, may still influence BMI and other metabolic traits. This interpretation is further supported by limited emerging evidence regarding *ATXN3* and metabolic traits. An independent study investigating pleiotropy between amyotrophic lateral sclerosis (ALS) and obesity-related traits identified rs978220, an intronic variant of *ATXN3*, as being shared between ALS and 11 obesity-related traits [25]. The authors suggested that *ATXN3* may contribute to both conditions through its role in protein processing, transport, and metabolic regulation, pathways in which abnormal protein metabolism has been implicated [25]. Although *ATXN3* is primarily associated with neurological conditions, and evidence linking it to obesity remains limited, our findings align with the possibility that trinucleotide repeats in this gene may influence obesity-related traits and further underscore the interconnected nature of brain-associated genetic mechanisms and metabolic regulation. Nevertheless, the functional impact of these repeats on BMI remains unclear, and their benign classification precludes interpreting them as direct drivers of the phenotype. Therefore, the role of *ATXN3* in obesity should be considered preliminary and hypothesis-generating, requiring validation in larger cohorts and functional studies.

### 4.2. Previously Reported Genetic Variants Related to Obesity Development

WES analysis identified two previously reported heterozygous non-synonymous variants of *ADIPOQ* and *SH2B1*, both of which have been associated with obesity and related metabolic conditions (Table 3).

A missense p.(Gly84Arg) rs199646033 variant of the adiponectin, C1Q, and collagen domain-containing (*ADIPOQ*) gene was detected in HP041 in family #2 with a paternal inheritance. Adiponectin, an adipocyte-secreted hormone involved in metabolic regulation, has been linked to obesity, abnormal cholesterol levels, and T2DM, with reduced circulating levels frequently observed in individuals with obesity [22,26,27,28]. Consistent with prior research, the *ADIPOQ* p.(Gly84Arg) variant identified in our cohort may contribute to obesity-related metabolic dysfunction. Previous studies in Japanese and French populations associated this variant with obesity and T2DM and suggested that substitutions affecting glycine residues within the conserved collagen domain may disrupt the triple-helix structure of adiponectin, impairing its biological function [29,30]. In our cohort, the presence of diabetes mellitus in the father (HP043) and insulin resistance in the index case (HP041) may support a similar metabolic effect.

*SH2B1* is a well-established regulator of body weight, and damaging variants of this gene have been associated with hyperphagia, severe early-onset obesity, and insulin resistance [31,32]. Additionally, CNVs involving the chr16p11.2 region harboring SH2B1 have been linked to increased BMI and obesity, further supporting its role in metabolic regulation [31,32,33]. The rare p.(Ser616Pro) heterozygous variant identified in family #3 is located within the functional SH2 domain and has been predicted to be damaging by *in silico* tools. Previous functional studies have suggested that this variant may alter leptin and insulin signaling and impair neuronal function involved in weight regulation, and it has also been reported in individuals with early-onset obesity [33,34].

Overall, the previously reported variants identified for *ADIPOQ* and *SH2B1* are biologically plausible candidates supported by prior research; however, further functional studies and larger cohorts are needed to clarify their contribution to obesity phenotypes in our families.

### 4.3. Novel Genetic Variants Related to Obesity Development

In addition to previously reported variants, our study identified novel genetic variants of genes associated with obesity and metabolic regulation. The familial segregation patterns observed in our cohort provide supportive evidence for their potential relevance; however, these findings remain hypothesis-generating and require validation in larger cohorts and functional studies to clarify their contribution to obesity.

Two candidate genetic variants were identified in family #3. The first one is a novel stop-gained variant p.(Gln404Ter) rs779673107 of the Ankyrin Repeat and Kinase Domain-Containing 1 (*ANKK1*) gene, which was detected in HP069, her mother and sister from family #3. *ANKK1* is closely linked to dopamine signaling pathways, particularly through its interaction with dopamine receptor type 2 (DRD2), which play important roles in food intake regulation and reward-related behaviors [22]. Genetic alterations involving *ANKK1* and *DRD2* have been associated with obesity, eating disorders, and addictive behaviors [35,36]. Moreover, the Taq1A rs1800497 p.(Glu713Lys) polymorphism of *ANKK1* has been widely associated with obesity and generates the A1 allele through a non-conservative amino acid substitution in exon 8. Reduced ANKK1 activity linked to this allele has been associated with obesity and is further supported by obese phenotypes observed in Ankk1 knockout mice [37,38,39]. The novel p.(Gln404Ter) variant identified in family #3 is located in close proximity to this polymorphism and is predicted to produce a truncated protein affecting a highly conserved region (GERP score: 4.69). An additional novel p.(Ile281Val) (rs758115329) variant of Neuronal Growth Regulator 1 (*NEGR1*) was identified in HP069 and segregated with affected family members, except for the mother. *NEGR1* is involved in neuronal regulation of feeding behavior and energy balance and has been consistently associated with obesity and increased BMI in both human genetic studies and animal models [22,40,41,42,43,44]. Notably, variants affecting *NEGR1* have been linked to altered food intake regulation, while Negr1 knockout models have demonstrated increased fat mass, adipose tissue expansion, and metabolic disturbances [41,42,43,44]. Supported by prior research and familial segregation patterns, the novel variants identified for *ANKK1* and *NEGR1* may represent candidate contributors to the obesity phenotype observed in family #3.

Genetic analyses in family #1 revealed novel variants of genes with indirect links to obesity-related pathways. One of these was a heterozygous p.(Tyr962His) variant of ATP Binding Cassette Subfamily B Member 1 (*ABCB1*), which showed paternal segregation. *ABCB1* encodes P-glycoprotein (P-gp), a transmembrane transporter involved in the efflux of xenobiotics and endogenous compounds, including hormones and lipids, across multiple tissues such as the blood–brain barrier, liver, intestine, and kidneys [22,45]. Previous studies have linked *ABCB1* polymorphisms and reduced gene expression to obesity-related metabolic alterations, including abnormal weight gain, elevated fasting glucose levels, and altered lipid metabolism [46,47,48]. Consistently, P-gp-deficient mice have demonstrated significantly greater weight gain compared with wild-type controls under identical dietary conditions [49]. The second novel missense Glycogen Synthase Kinase 3β (*GSK3β*) p.(Asn374Ser) (rs1347336963) variant was identified in HP009 and his father and was found to be extremely rare across population databases. *GSK3β* is a highly conserved intracellular serine/threonine kinase that plays a critical role in glucose metabolism, insulin signaling, and energy homeostasis through the regulation of glycogen synthesis [22]. Beyond glucose regulation, *GSK3β* has also been implicated in lipid accumulation, adipogenesis, leptin signaling, and mitochondrial energy metabolism, highlighting its broader role in obesity-related pathways [50,51]. Notably, its expression in adipose tissue has been shown to correlate with body fat distribution, further supporting its relevance in obesity phenotypes [52,53]. Taken together, the novel variants identified for *ABCB1* and *GSK3β* have not been previously reported, likely due to their extreme rarity in population databases. However, the established links of these genes to obesity-related pathways, their segregation patterns consistent with the phenotype observed in family #1, and supporting bioinformatic evidence including evolutionary conservation (GERP scores: 5.79 and 4.77) and predicted deleteriousness (CADD scores: 25.6 and 24.6) suggest that they may represent meaningful candidate variants for further investigation in obesity.

In family #2, WES analysis identified a novel missense 2-oxoglutarate dehydrogenase (*OGDH*) variant, p.(Ala656Val) (rs777352026), present in the heterozygous state in HP041, his mother, and two siblings. *OGDH* encodes a mitochondrial matrix enzyme involved in the tricarboxylic acid (TCA) cycle, a key pathway regulating cellular energy metabolism and redox homeostasis [22]. Previous studies have linked reduced OGDH activity to metabolic dysfunction; decreased enzymatic activity has been associated with impaired oxidative capacity in skeletal muscle and obesity-related metabolic alterations [54]. Supporting this role, Ogdh^+^/^−^ mouse models have demonstrated significantly greater weight gain, particularly under a high-fat diet, accompanied by reduced OGDH expression and liver dysfunction [55]. Notably, unlike the variants identified in other families, the *OGDH* p.(Ala656Val) variant demonstrated consistently strong bioinformatic support, predicted as disease-causing by MutationTaster, deleterious by SIFT, and probably damaging by PolyPhen-2. This interpretation is further supported by high evolutionary conservation (GERP score: 5.13) and a high predicted deleteriousness (CADD score: 31), supporting its candidacy as a potential contributor to the obesity phenotype observed in family #2.

## 5. Conclusions

Identifying genetic factors potentially associated with obesity phenotypes in each family may be valuable for improving future genetic counseling strategies and advancing our understanding of familial obesity. In this exploratory study, we identified rare and novel variants in genes involved in metabolic and obesity-related pathways, which were supported by familial segregation patterns and prior research. However, the functional significance and clinical relevance of these variants remain uncertain. Therefore, our findings should be considered hypothesis-generating and provide a foundation for future functional studies and larger population-based investigations to further explore the genetic basis of familial obesity and related metabolic conditions.

## 6. Study Limitations

This study has several limitations. We lacked detailed functional analyses to clarify the biological effects of the identified variants and their potential disease-related mechanisms. In addition, the relatively small sample size limits the generalizability of our findings, particularly given the polygenic nature of obesity, where the presence of multiple variants across genes with diverse biological functions is not unexpected. In our cohort, the identified genes did not converge on a single biological pathway, limiting our ability to infer specific gene–gene interactions. To partially address sample size limitations, family members were pooled, which enabled the identification of the shared rs3356231 variant that may reflect population-specific genetic variation. Therefore, these findings should be interpreted cautiously given the exploratory nature of this study.

## Figures and Tables

**Table 1:** Details of data quality and filtering results revealed by WES analysis.

Quality Metrics of WES Data for HP009 Family #1		Quality Metrics of WES Data for HP041 Family #2		Quality Metrics of WES Data for HP069 Family #3	
Total number of reads aligned	46.5 M	Total number of reads aligned	46.4M	Total number of reads aligned	38.0 M
Average depth (%)	100.13	Average depth (%)	100.13	Average depth (%)	81.23
% Targets with 50× coverage	99.58	% Targets with 50× coverage	98.58	% Targets with 50× coverage	99.44
Total number of annotations	201.8 K	Total number of annotations	204.9 K	Total number of annotations	193.6 K
Total number of variants	45,411	Total number of variants	44,636	Total number of variants	44,299
Variants of candidate obesity genes	5705	Variants of candidate obesity genes	5497	Variants of candidate obesity genes	5588
(Supplementary Table S1)		(Supplementary Table S1)		(Supplementary Table S1)	
Number of pathogenic variants *	2	Number of pathogenic variants *	1	Number of pathogenic variants *	0
Number of likely pathogenic variants *	6	Number of likely pathogenic variants *	2	Number of likely pathogenic variants *	3
Number of variants of uncertain significance (VUS) *	31,437	Number of variants of uncertain significance (VUS) *	32,368	Number of variants of uncertain significance (VUS) *	29,104
Homozygous variants	15,223	Homozygous variants	16,406	Homozygous variants	15,004
Heterozygous variants	30,186	Heterozygous variants	28,248	Heterozygous variants	29,310
Variant filtering for MAF ≤ 0.01	4282	Variant filtering for MAF ≤ 0.01	4402	Variant filtering for MAF ≤ 0.02	4089

* Pathogenicity determined in accordance with ACMG guidelines. Abbreviations: M: million; K: thousand; MAF: minor allele frequency.

**Table 2:** Details of the shared obesity-related variants among families.

Patient Code	Zygosity	Gene	Variant	Amino Acid Change	dbSNP ID	Impact	MAF	Pathogenicity ACMG/SEQ	ClinVar
**HP009 Family #1**	Homozygous	***ATXN3***	ENST00000393287.5:c.915_916ins (CAGCAGCAGCAGCAGCAGCAGCAGCAG)	p.(Gln297_Gln305dup)	rs193922928	Inframe Insertion	NA	VUS+/VUS+	Reported (B) for MJD
**HP041 Family #2**	Heterozygous								
**HP069 Family #3**	Heterozygous								

Abbreviations: MAF: minor allele frequency; NA: not applicable; VUS: variant of uncertain significance; B: benign; MJD: Machado–Joseph disease; ACMG: American College of Medical Genetics and Genomics.

**Table 3:** Details of the detected obesity-related variants with confirmed family segregation.

Patient Code	Gene	Variant	Amino Acid Change	dbSNP ID	Impact	Zygosity	MAF	Pathogenicity ACMG/SEQ	ClinVar	Reference PMID
**Family#2**	***ADIPOQ***	ENST00000412955.2:c.250G>A	p.[Gly84Arg]	rs199646033	Missense	Heterozygous	<0.01	LP/VUS	Not reported	12878598 11812766 12354786
**Family#3**	***SH2B1***	ENST00000322610.8:c.1846T>C	p.[Ser616Pro]	rs142515048	Missense	Heterozygous	<0.01	LP/LP	Reported [LP] *	29631267 31439647

* Variant was reported in the ClinVar database for conditions of premature ovarian failure and 16p11.2 Microdeletion Syndrome. Abbreviations: MAF: minor allele frequency; VUS: variant of uncertain significance; LP: likely pathogenic; ACMG: American College of Medical Genetics and Genomics.

**Table 4:** Details of the *novel* candidate variants in obesity-related genes with confirmed family segregation.

Patient Code	Gene	Variant	Amino Acid Change	dbSNP ID	Impact	MAF	Pathogenicity ACMG/SEQ	ClinVar	Mutation Taster	SIFT	Poly Phen2	CADD Score *	GERP Score **
**Family#1**	***ABCB1***	ENST00000265724.3:c.2884T>C	p.[Tyr962His]	-	Missense	NA	VUS/VUS	Not reported	Disease Causing	Tolerated	Probably Damaging	25.6	5.79
	***GSK3B***	ENST00000316626.5:c.1121A>G	p.[Asn374Ser]	rs1347336963	Missense	<0.01	VUS/VUS	Not reported	Disease Causing	Deleterious	Benign	24.6	4.77
**Family#2**	***OGDH***	ENST00000222673.5:c.1967C>T	p.[Ala656Val]	rs777352026	Missense	<0.01	VUS/LP	Not reported	Disease Causing	Deleterious	Probably Damaging	31	5.13
**Family#3**	***NEGR1***	ENST00000357731.5:c.841A>G	p.[Ile281Val]	rs758115329	Missense	<0.01	VUS/VUS	Not reported	Disease Causing	Tolerated	Benign	17.80	5.83
	***ANKK1***	ENST00000303941.3:c.1210C>T	p.[Gln404Ter]	rs779673107	Stop Gained	<0.01	VUS/VUS	Not reported	Disease Causing	NA	NA	37	4.69

* Variants with CADD > 20 are predicted to be among the 1.0% most deleterious possible substitutions in the human genome. ** GERP score is a measure of sequence conservation across multiple species. A score greater than 2 can be considered evolutionary-constrained. dbSNP [https://www.ncbi.nlm.nih.gov/snp/, accessed on 4 February 2026]. PolyPhen 2 [www.genetics.bwh.harvard.edu/pph2/, accessed on 4 February 2026]. Abbreviations: MAF: minor allele frequency; VUS: variant of uncertain significance; LP: likely pathogenic; NA: not applicable; ACMG: American College of Medical Genetics and Genomics.

## Data Availability

The datasets generated and/or analyzed during the current study are not publicly available due to the Personal Data Protection Law (KVKK) in Türkiye, but are available from the corresponding author on reasonable request.

## Supplementary Materials

The following supporting information can be downloaded at: https://www.mdpi.com/article/10.3390/genes17050530/s1, Table S1: List of candidate genes for obesity.
