# Supplementary material for: Identifying Genetic Factors Contributing to Non-Syndromic Early-Onset Childhood Obesity Utilizing Whole-Exome Sequencing in Consanguineous Families
Source: Genes (Basel). 2026 Apr 29;17(5):530. doi: 10.3390/genes17050530 (PMC13205815; doi:10.3390/genes17050530)
Supplement: Supplementary file 1 [file genes-17-00530-s001.zip › genes-4286276-supplementary.pdf]

Supplementary Table S1: List of candidate genes for obesity

ACP1	Acid phosphatase 1
ADCY3	Adenylyl cyclase 3
ADD1	Alpha-adducin
ADIPOQ	Adiponectin
ADRA2A	Adrenergic receptor alfa-2A
ADRA2B	Adrenergic receptor alfa-2B
ADRB1	Adrenergic receptor ß-1
ADRB2	Adrenergic receptor ß-2
ADRB3	Adrenergic receptor ß-3
AGRP	Agouti related neuropeptide
BDNF	Brain-derived neurotrophic factor
CARTPT	Cocaine- and amphetamine-regulated transcript prepropeptide
CCK	Cholecystokinin
CEP19	Centrosomal protein 19 kDa
CFD	Complement factor D
CPE	Carboxypeptidase E
DRD2	Dopamine receptor D2
DYRK1B	Dual specificity tyrosine phosphorylation regulated kinase 1B
ENPP1	Ectonucleotide pyrophosphatase/phosphodiesterase 1
FTO	Fat mass- and obesity-associated gene
GHRL	Ghrelin and obestatin prepropeptide
GIPR	Gastric inhibitory polypeptide receptor
HDAC8	Histone deacetylase 8 kDa
IGSF1	Immunoglobulin superfamily member1
INS	Insulin
INSIG2	Insulin-induced gene2
IRS-1	Insulin receptor substrate 1
KSR2	Kinase suppressor of ras 2
LEP	Leptin
LEPR	Leptin receptor
LIPE	Hormone sensitive lipase
MC3R	Melanocortin 3 receptor
MC4R	Melanocortin 4 receptor
MRAP2	Melanocortin 2 receptor accessory protein 2
NAMPT	Nicotinamide phosphoribosyltransferase
NEGR1	Neuronal growth regulator 1
NPY	Neuropeptide Y
NR0B2	Nuclear receptor subfamily 0 group B member2
NR3C1	Nuclear receptor subfamily 3 group C member1
NTRK2	Neurotrophic tyrosine kinase receptor type 2
PCSK1	Proprotein conertase subtilisin/kexin type 1
POMC	Propiomelanocortin
PPARG	Peroxisome proliferative-activated receptor gamma

PTPN1	Tyrosine-protein phosphatase non-receptor type 1
PYY	Peptide tyrosine tyrosine
RETN	Resistin
SDC3	Syndecan 3
SH2B1	SH2B adaptor protein 1
SIM1	Single-minded (Drosophila) homologue 1
SLC22A1	Solute carrier family 22 member 1
SLC2A4	Solute carrier family 2 member 4
SREBF1	Sterol regulatory element-binding transcription factor 1
TMEM18	Transmembrane protein 18 kDa
TUB	Tubby bipartite transcription factor
UCP1	Uncoupling protein 1
UCP2	Uncoupling protein 2
UCP3	Uncoupling protein 3

Supp Table S1: The list of obesity-related candidate genes used in variant prioritization on bioinformatic analysis. These genes are known to be directly related to obesity in patients. Disruptions in these genes, associated pathways, or interacting genes usually induce obesity. **Note:** The candidate gene list presented in Supplementary Table 1 was compiled based on the obesity-associated genes available at the time of analysis using databases such as OMIM, ClinVar, relevant literature sources, and previously reported obesity-related gene panels. This list was not intended to be exhaustive or definitive, and additional candidate genes may be identified as databases are updated and new evidence emerges.
